# Study on the Preoperative and Postoperative Levels of Serum Lactate to Estimate Morbidity and Mortality in Cases of Bowel Perforation at a Tertiary Care Hospital in Jharkhand, India

**DOI:** 10.7759/cureus.84748

**Published:** 2025-05-24

**Authors:** Samir Toppo, Kumar Gaurav, Ranjana Mondal, Krishan Kumar, Kamlesh Kumar

**Affiliations:** 1 General Surgery, Rajendra Institute of Medical Sciences, Ranchi, IND

**Keywords:** anastomotic leak after gastrointestinal surgery, gastro-intestinal perforation, postoperative mortality, prolonged length of hospital stay, serum lactate, spontaneous bowel perforation, surgical site occurrences, traumatic small bowel perforation

## Abstract

Introduction

Bowel perforation is a common emergency with high morbidity and mortality. The patient presented with acute abdomen, abdominal distention, obstipation, and frequent gas under the diaphragm on radiogram. Arterial blood gas (ABG) is one of the earliest investigations done in cases of suspected bowel perforation in the Emergency Room (ER), and includes serum lactate.

Objective

This study evaluates the role of sequential monitoring of serum lactate levels while ensuring a consistent timing of measurements and finding associations with prolonged hospital stay, surgical site occurrence, anastomotic leak, and mortality outcomes.

Methods

The study included 72 patients who presented to the healthcare facility within 72 hours of the onset of symptoms or occurrence. Continuous ABG was done before surgery and at POD 1, 2, 3, 5, and 7, and various parameters like age, sex, comorbidities, duration of presentation, site of perforation, prolonged hospital stay, surgical site occurrence, anastomotic leak, and mortality were noted in the form of tables.

Results

Analysis revealed that the mean serum lactate levels were generally higher in patients with prolonged hospital stays as compared to those without; however, there was no statistical significance at any time point. For surgical site occurrences (SSOs), there was no statistically significant difference in lactate levels between the two groups at Pre-Op, POD1, and POD2; however, significant differences were observed since POD3. For anastomotic leak, significant differences were observed at POD2. And for mortality, preoperatively, the mean lactate level in the mortality group was 5.91 mmol/L and 2.74 mmol/L in the non-mortality group, and this was statistically significant in all time periods.

Conclusion

Elevated serum lactate levels were consistently associated with adverse postoperative outcomes, including prolonged hospital stay, SSOs, anastomotic leaks, and mortality, with significance observed particularly from POD2 onward. Although not all differences were statistically significant, the trends suggest a meaningful clinical correlation. Routine lactate monitoring may serve as an important tool for the early detection of complications and risk stratification.

## Introduction

Bowel perforation, a common emergency with high morbidity and mortality, requires urgent surgical intervention when there is a tear in the wall of the bowel. This can lead to the leakage of intestinal contents into the abdominal cavity, which may cause peritonitis, sepsis, and even mortality. Bowel perforation can result from a variety of causes, which can be due to external trauma or underlying medical conditions. Patients with bowel perforation present with acute abdomen, abdominal distention, and obstipation, and frequently have gas under the diaphragm on the radiogram. Surgical intervention is necessary in almost all cases except stable cases with a sealed perforation.

Following patients’ admission, we conducted arterial blood gas analysis and routine investigation (including serum lactate). Arterial blood gas (ABG) is one of the earliest investigations in cases of suspected bowel perforation in the ER and assesses a patient’s oxygenation, ventilation, and acid-base status.

Lactate levels are a surrogate marker for tissue perfusion and a powerful standalone predictor of prognosis in various situations. Elevated lactate levels are indicative of conditions such as bowel ischemia, shock, and sepsis. Lactate levels have been more specific than leukocyte count in diagnosing abdominal sepsis [1].

In this study, we aimed to evaluate the role of sequential monitoring of serum lactate levels, considering recent advancements in available diagnostic tools while ensuring consistent timing of measurements. This study incorporates both morbidity and mortality outcomes, offering a comprehensive understanding of the clinical impact.

## Materials and methods

We conducted this single hospital-based analytic cross-sectional study over a period of 24 months in the Department of General Surgery at the Rajendra Institute of Medical Sciences (RIMS) in Ranchi, Jharkhand, India, and included 72 patients diagnosed with bowel perforation.

Inclusion criteria

The following types of patients were included: 1. All patients with bowel perforation diagnosed at RIMS, Ranchi; 2. Patients aged ≥ 18 years and ≤ 75 years; 3. Either sex; 4. Presenting to the healthcare facility within 72 hours of the onset of symptoms or occurrence.

Exclusion criteria

The following types of patients were excluded: 1. Pregnant patients; 2. Patients with known medical conditions, including diabetes mellitus, hepatic failure, renal failure, malignancy, thiamine deficiency, and cardiovascular diseases, as well as patients with additional comorbidities that could further complicate their clinical management; 3. Patients receiving treatments including anti-tubercular therapy, antiretroviral therapy, beta-agonists, biguanides, salicylates, and bronchodilators such as salbutamol; 4. Patients who did not survive throughout the designated seven-day trial period; 5. Patients who either declined to provide consent, withdrew from the trial, or were transferred to another facility.

We conducted the study on all patients diagnosed with bowel perforation at RIMS, Ranchi, who met predefined eligibility criteria to ensure an accurate and comprehensive evaluation. We explained the purpose, procedures, risks, and benefits to the patients and/or their attendants, and we obtained informed consent only after they thoroughly understood their participation and voluntarily agreed to it. We performed a detailed medical history and clinical examination, supported by relevant radiological investigations and ABG analysis for a complete assessment. We investigated complications, such as surgical site infections, anastomotic leaks, and prolonged hospital stays, due to their potential impact on recovery. We collected ABG samples before surgery and at 24, 48, 72, 120, and 168 hours postoperatively. We monitored patients until the seventh postoperative day and followed up until discharge to document outcomes, including surgical site occurrences (SSOs), prolonged hospitalization, and mortality. We defined a prolonged hospital stay as one exceeding 14 days, and SSOs included a range of wound-related complications beyond surgical site infections. For mortality tracking, we collected contact information at discharge and conducted follow-ups via outpatient visits and phone calls one month post-surgery.

We coded and entered the collected data in a Microsoft Excel sheet (Microsoft Corporation, Redmond, WA, US), further analyzed them using IBM SPSS Statistics version 29.0 software (IBM Corp., Armonk, NY, US), and presented the results in tabular form and percentage-wise graphical format. We generated a data master sheet for the variable study. We expressed the qualitative data in proportions and percentages, and the quantitative data in means. We used an unequal variances t-test to find the significance of study parameters on a categorical scale between two or more groups. We considered statistical significance as a p-value of less than 0.05.

## Results

We noted serum lactate levels just at the time of presentation and postoperatively at 24 hours (postoperative day (POD) 1), 48 hours (POD 2), 72 hours (POD 3), 120 hours (POD 5), and 168 hours (POD 7). We then followed up patients on POD 7 to assess for SSOs and anastomotic leaks, and after POD 7 till discharge for a prolonged hospital stay, with additional tracking for mortality until POD 28.

All 72 patients in this study underwent emergency surgery and satisfied the inclusion criteria. 51 (70.83%) were men and 21 (29.17%) were women, with a ratio of 2.4: 1 (M: F). The minimum age of patients included in this study was 18 years, the maximum age was 75 years. The average age was 41.3 years (average age in men = 41.8 years and 40.2 years in women). The age group 31-40 years had the highest number of patients (18 individuals, comprising 13 men and 5 women).

In this study, patients presented to the healthcare facility within 72 hours of the onset of symptoms or occurrence. Day 2 had the highest number of presentations, i.e., 44.44%, with a total of 32 individuals. Follow-up on day 3, i.e., 34.72%, had a total of 25 individuals, and the least presented on day 1, i.e., 20.83%, with 15 individuals.

All types (as per site of perforation) of bowel perforation were included in this study, but iatrogenic perforations were excluded. The stomach had the highest number of perforation cases, accounting for 50% of the total cases (36 individuals). Other sites, such as the ileum (20.83%) and appendix (11.11%), also had notable proportions, whereas sites such as the jejunum, transverse colon, cecum, and rectum had relatively lower percentages, ranging from 2.78% to 4.17%.

While comparing the day of presentation with the site of perforation, the stomach was the most affected site on Day 2, contributing 18 cases (56.25% of Day 2 cases). Whereas, ileal perforation saw a notable increase on Day 2 (7 cases), with a total of 15 cases, making it the second most frequent site, and appendicular perforation being consistent throughout the days, with 3 cases on Day 1, 3 on Day 2, and 2 on Day 3, totaling 8 cases. Duodenum, jejunum, cecum, transverse colon, and rectal perforation showed a lower frequency of cases overall, with some fluctuations, such as the cecum having three cases on Day 3.

**Figure 1:**
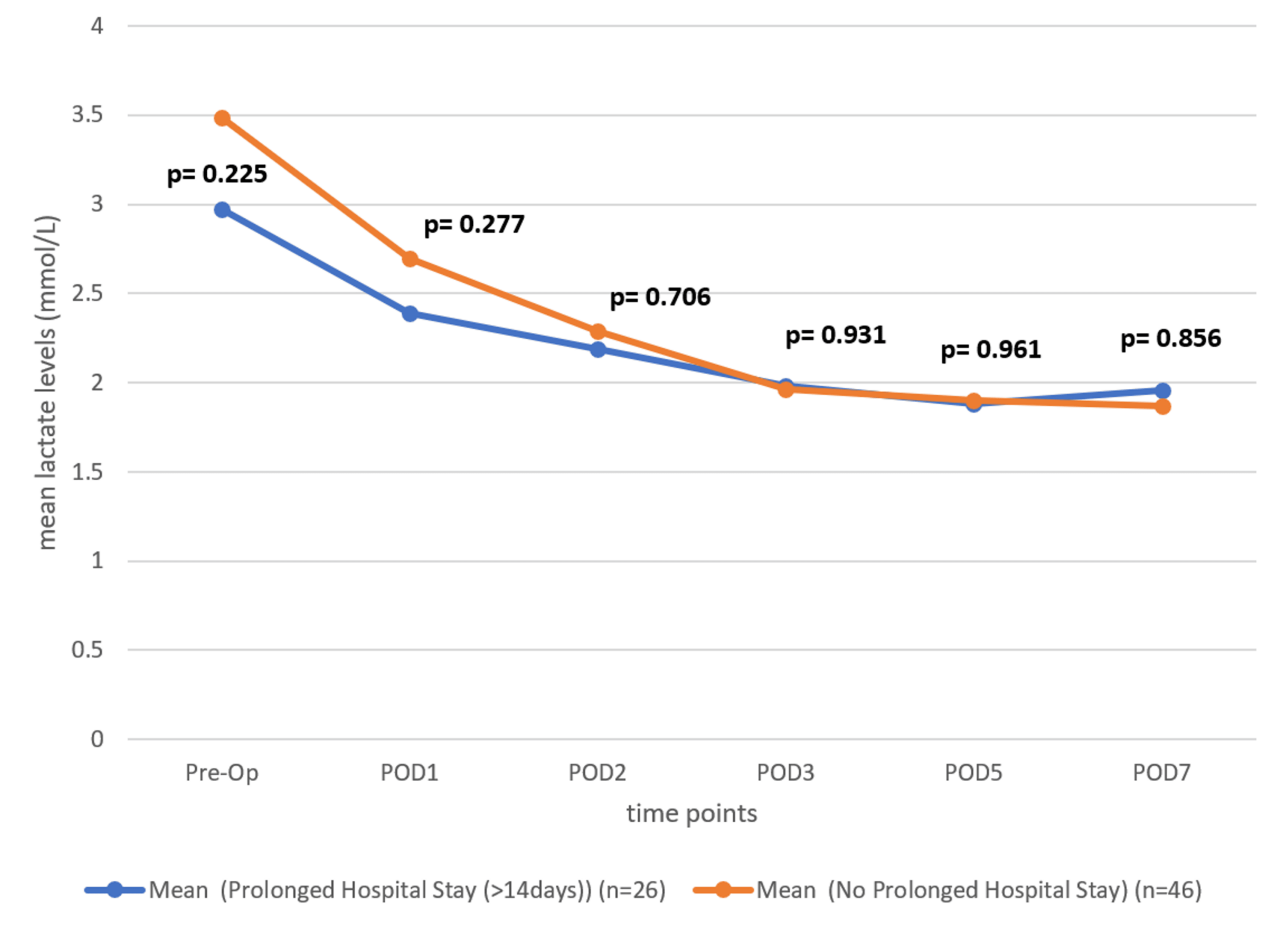
The line diagram illustrates the trend of mean serum lactate levels over the preoperative and postoperative periods at POD1, POD2, POD3, POD5, and POD7 for patients without and with prolonged hospital stays (>14 days). (The blue line represents patients with prolonged hospital stays, while the orange line represents those without prolonged hospital stays).

To evaluate the relation between the preoperative and postoperative levels of serum lactate with prolonged hospital stay (>14 days), first, we excluded those patients who died before POD 14. We conducted an unequal variances t-test at PODs 1, 2, 3, 5, and 7 to compare serum lactate levels between patients who experienced prolonged hospital stays and those who did not. The analysis revealed that the mean serum lactate levels were generally higher in patients with prolonged hospital stays as compared to those without. However, the differences observed were not statistically significant at any time point (i.e., p > 0.05 for all comparisons). For instance, on POD 1, the mean serum lactate level in the prolonged stay group was 2.3881 mmol/L, while in the non-prolonged stay group, it was 2.6943 mmol/L (with t = −1.10 and p = 0.225). Similarly, on POD 5, the means were 1.8825 mmol/L and 1.8990 mmol/L, respectively (with t = −0.049 and p = 0.961) (Table 1 and Figure 1).

**Table 1:** Comparison of serum lactate levels between patients with prolonged hospital stay (>14 days) and no prolonged hospital stay across preoperative and postoperative periods

Periods	Mean (mmol/L) (Prolonged Hospital Stay (>14 days)) (n=26)	Mean (mmol/L) (No Prolonged Hospital Stay) (n=46)	t-value	p-value
Pre-Op	2.9688	3.4858	-1.23	0.225
POD1	2.3881	2.6943	-1.10	0.277
POD2	2.1913	2.2895	-0.38	0.706
POD3	1.9844	1.9605	0.088	0.931
POD5	1.8825	1.8990	-0.049	0.961
POD7	1.9594	1.8690	0.184	0.856

**Figure 2:**
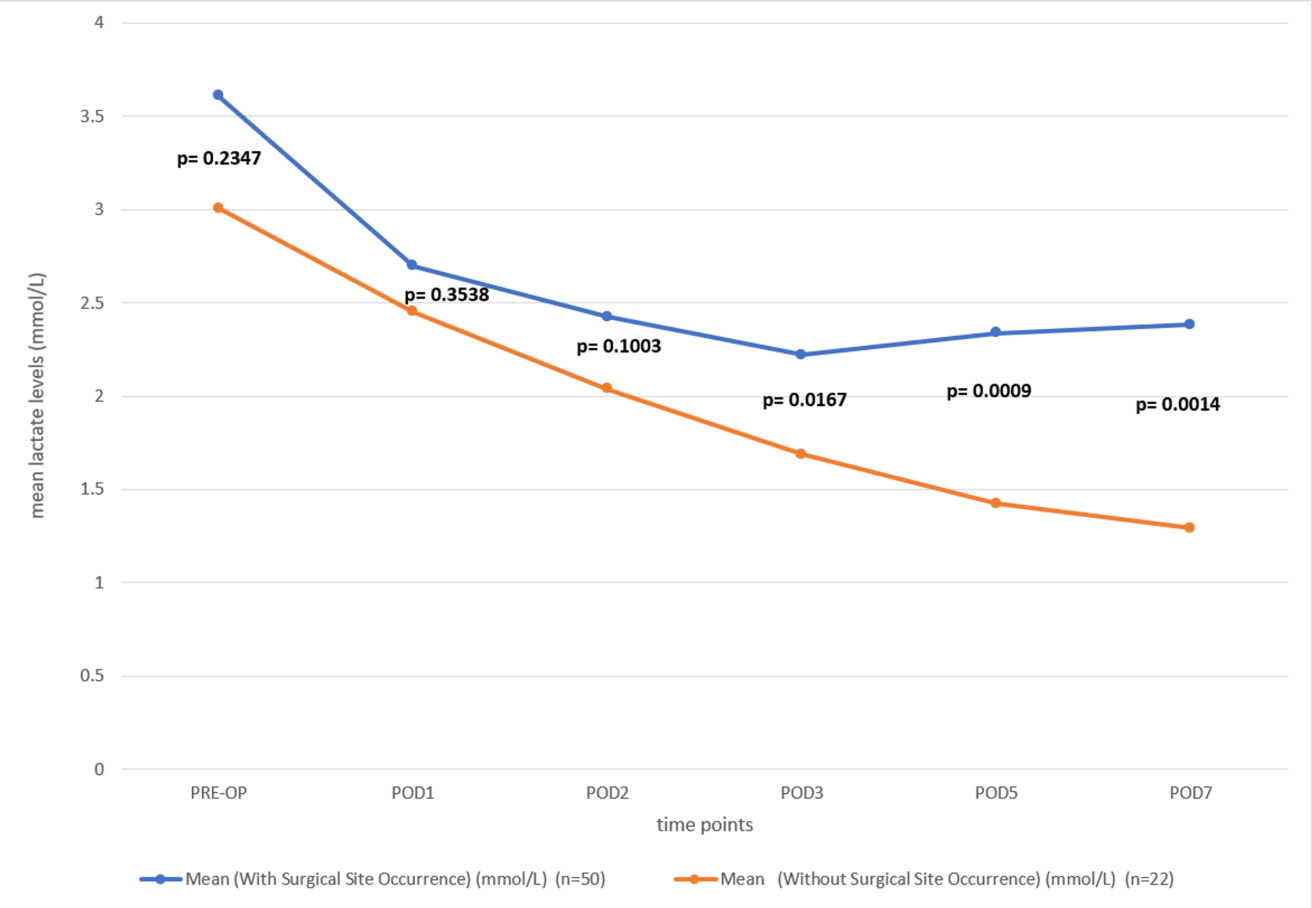
The line diagram illustrates the trend of mean serum lactate levels over preoperative and postoperative periods at POD 1, POD 2, POD 3, POD 5, and POD 7 for patients with and without surgical site occurrences Both groups exhibit a decline in lactate levels post-operation, particularly during the first few days (POD 1 to POD 3). By POD 5 and POD 7, there is a noticeable divergence, with the "Occurrence" group maintaining higher lactate levels, consistent with the significant differences observed in the statistical analysis. (The blue line represents patients with a surgical site occurrence, while the orange line represents those without a surgical site occurrence).

To evaluate the relation between preoperative and postoperative levels of serum lactate with surgical site occurrences, we conducted an unequal variances t-test to compare the serum lactate levels between patients with and without SSOs at the preoperative period and PODs 1, 2, 3, 5, and 7. The results indicate that there was no statistically significant difference in lactate levels between the two groups at preoperative (p = 0.2347), POD 1 (p = 0.3538), and POD 2 (p = 0.1003). However, we observed significant differences at POD 3 (p = 0.0167), POD 5 (p = 0.0009), and POD 7 (p = 0.0014), with higher lactate levels in the SSO group as compared to the non-SSO group. These findings suggest that elevated lactate levels in the later postoperative period may be associated with the occurrence of surgical site complications. (Table 2 and Figure 2).

**Table 2:** Comparison of serum lactate levels between patients with surgical site occurrence and without surgical site occurrence across preoperative and postoperative periods

Period	Mean (With Surgical Site Occurrence) (mmol/L) (n=50)	Mean (Without Surgical Site Occurrence) (mmol/L) (n=22)	T-Value	P-Value
PRE-OP	3.61	3.007	1.208	0.2347
POD1	2.697	2.452	0.936	0.3538
POD2	2.425	2.039	1.673	0.1003
POD3	2.221	1.686	2.468	0.0167
POD5	2.338	1.423	3.567	0.0009
POD7	2.383	1.293	3.415	0.0014

**Figure 3:**
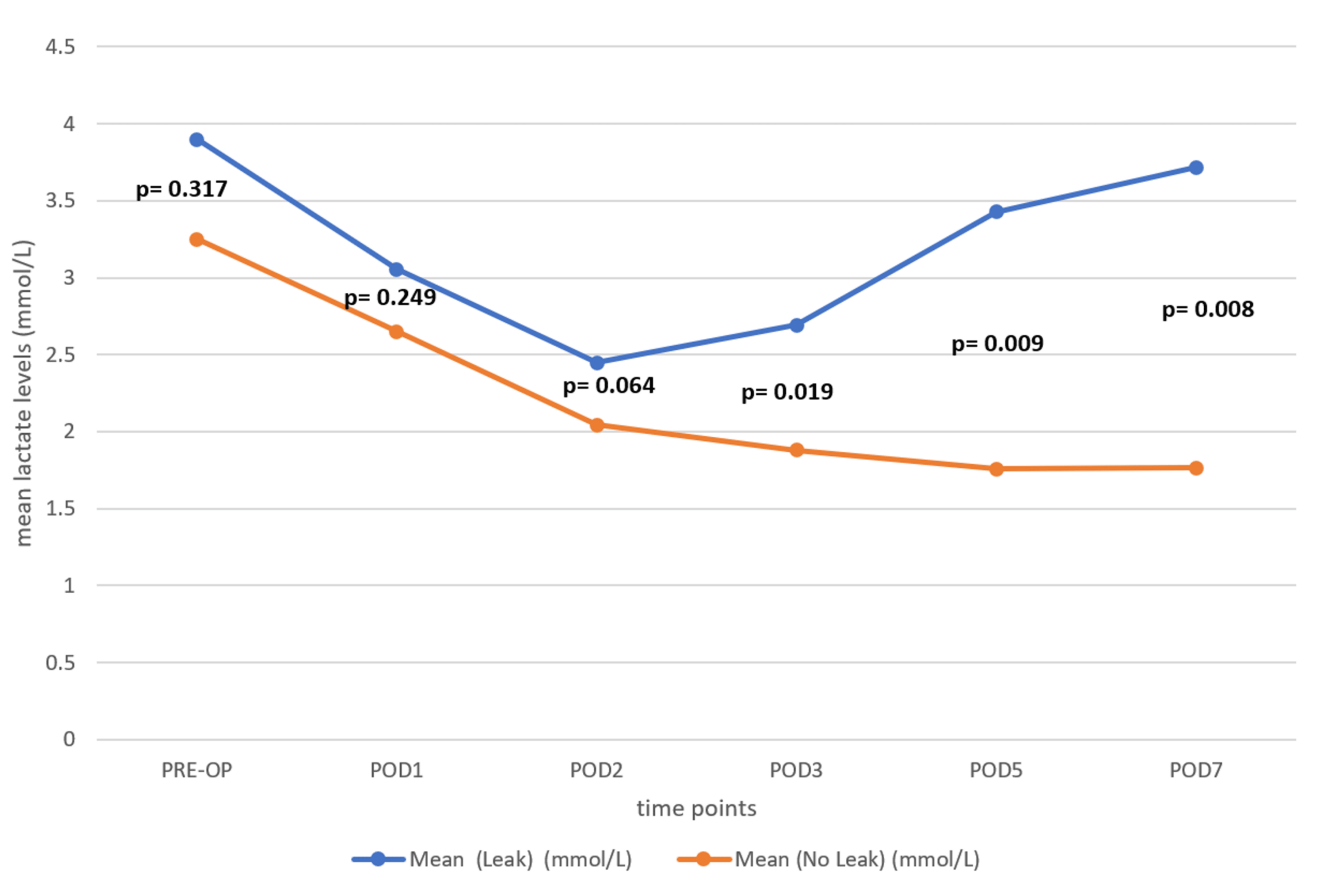
The line diagram shows the trend of mean values over different postoperative days for patients with and without an anastomotic leak, having divergence from POD2 and peak difference at POD5 The blue line represents patients with an anastomotic leak, while the orange line represents those without an anastomotic leak.

To find the association with the anastomosis leak, we conducted an unequal variances t-test to compare the mean values between patients with and without anastomotic leaks during the preoperative period and PODs 1, 2, 3, 5, and 7. The results revealed no significant differences in mean values during the preoperative (p = 0.317) and POD 1 (p = 0.249) period, whereas on POD 2, the difference in mean values approached statistical significance (p = 0.064), suggesting potential early divergence and significant differences from POD 3 onwards, with the mean biomarker levels being consistently higher in the leak group compared to the no-leak group. Specifically, we found significant differences at POD 3 (p = 0.019), POD 5 (p = 0.009), and POD 7 (p = 0.008), with the leak group showing markedly elevated levels. These findings suggest that rising biomarker levels from POD 3 may indicate anastomotic leaks, highlighting a potential time window for early detection and intervention (Table 3 and Figure 3)

**Table 3:** Comparison of serum lactate levels between patients with an anastomotic leak and those without an anastomotic leak across the preoperative and postoperative periods

Periods	Mean (Leak) (mmol/L) (n=11)	Mean (No Leak) (mmol/L) (n=61)	t-statistic	p-value
PRE-OP	3.899	3.248	1.062	0.317
POD1	3.054	2.649	1.218	0.249
POD2	2.449	2.045	2.055	0.064
POD3	2.693	1.88	2.941	0.019
POD5	3.429	1.759	3.483	0.009
POD7	3.717	1.765	3.489	0.008

We also performed an unequal variances t-test to compare lactate levels between the mortality and non-mortality groups during the preoperative period and PODs 1, 2, 3, 5, and 7. The mean lactate levels were consistently higher in the mortality group as compared to the non-mortality group at all measured time points. The differences were statistically significant at each interval, with p-values < 0.05 throughout.

**Figure 4:**
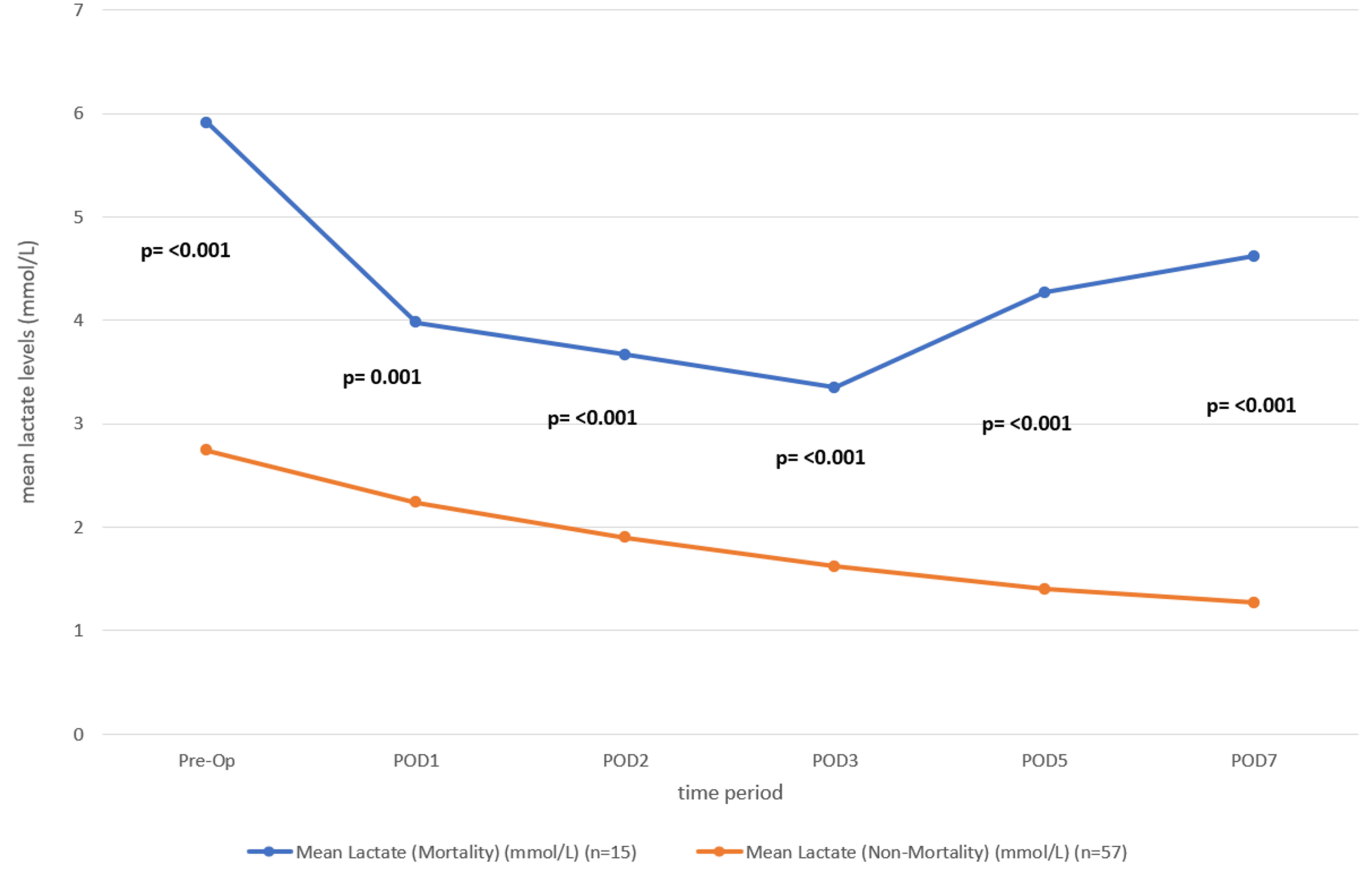
The line diagram representing the mean lactate levels over time for the mortality and non-mortality groups The blue line represents the mortality group, and the orange line represents the non-mortality group. The trend shows that lactate levels tend to decrease over time in both groups, but the mortality group consistently exhibits higher lactate levels.

Preoperatively, the mean lactate level in the mortality group was 5.91 mmol/L, significantly higher than the 2.74 mmol/L observed in the non-mortality group (t = 5.12, p < 0.001). This trend persisted postoperatively, with mean lactate levels remaining elevated in the mortality group from POD 1 (3.98 vs. 2.24, p = 0.001) to POD 7 (4.62 vs. 1.27, p < 0.001). We observed the highest statistical significance on POD 5 and POD 7, suggesting a strong correlation between persistently elevated lactate levels and mortality risk (Table 4 and Figure 4).

**Table 4:** Comparison between mean lactate levels with mortality across the preoperative and postoperative periods

Time Point	Mean Lactate (Mortality) (mmol/L) (n=15)	Mean Lactate (Non-Mortality) (mmol/L) (n=57)	t-value	p-value
Pre-Op	5.91	2.74	5.12	<0.001
POD1	3.98	2.24	3.67	0.001
POD2	3.67	1.9	4.02	<0.001
POD3	3.35	1.62	3.88	<0.001
POD5	4.27	1.4	4.78	<0.001
POD7	4.62	1.27	5.21	<0.001

## Discussion

In this study, we aim to evaluate the role of preoperative and postoperative serum lactate levels in assessing the association between lactate levels and prolonged hospital stay, SSOs, and anastomotic leaks, and estimating mortality rates.

Serum lactate levels are routinely used to assess tissue perfusion and guide treatment in critically ill patients. Persistent lactate elevation is linked to high mortality and organ damage, with most studies focusing on mortality risk in critically ill patients rather than morbidity [2].

This study involved 72 emergency bowel perforation cases, with 70.83% comprising men with an average age of 41.3 years. The 31-40 age group was most affected (25%), but younger individuals were also at risk, with 28% of patients aged 18-30. Most patients presented within 72 hours, with the highest on Day 2 (44.44%). Delayed presentation, likely due to mild early symptoms or limited healthcare access, may have led to worse outcomes, especially for those presenting on Day 3.

Multiple researchers have cited the absence of serial blood lactate measurements as a limitation due to their retrospective design. To address this gap, we incorporated serial lactate monitoring as part of our methodology. Dettmer et al. found that patients with severe sepsis or septic shock who received serial lactate monitoring had a greater reduction in lactate levels, leading to lower 28-day mortality and more ventilator- and vasopressor-free days, thus supporting serial lactate monitoring as an effective tool for improving outcomes in the emergency department [3].

Due to variations in lactate production and elimination in critically ill patients, serial lactate measurements are recommended. However, the optimal timing for measuring lactate remains unclear because its interpretation is complicated by the complex pathophysiology [4]. Thus, we sought to address this by systematically measuring and recording serum lactate levels on fixed preoperative and postoperative days.

Hyperlactatemia after major surgery is often linked to tissue hypoperfusion and hypoxia, but can also result from disrupted glucose metabolism and altered insulin regulation. Elevated catecholamines during surgical stress further increase lactate by stimulating glycolysis and reducing clearance. These factors underscore the need for cautious interpretation of lactate levels postoperatively [5].

In this study, patients without prolonged hospital stays exhibited slightly higher mean serum lactate levels than patients with prolonged hospital stays, and the differences remained statistically insignificant at all time points (p > 0.05). These findings indicate that serum lactate levels alone are not a reliable predictor of prolonged hospital stay. Factors such as the patient’s preoperative condition, type of surgery performed, and any postoperative complications likely played a more significant role in determining the duration of the hospital stay.

However, Henry et al. found that elevated serum lactate levels were associated with a longer hospital stay, with a statistically significant p-value of 0.043 [6]. Similarly, Creagh-Brown et al., in a large cohort of 121,990 patients, reported that higher peak lactate levels within the first 24 hours post-surgery were strongly correlated with increased mortality and extended hospitalization, even when values remained within the normal range [7].

For association with SSO, we found no significant difference in lactate levels before surgery or on POD 1 and POD 2 (p > 0.05). However, by POD 3, significant differences emerged, with patients who developed SSO exhibiting higher lactate levels compared to those without SSO (p = 0.0167). The statistical significance increased further on POD 5 (p = 0.0009) and POD 7 (p = 0.0014). This suggests that elevated lactate levels in the later postoperative period may be associated with surgical site complications. These findings highlight the potential usefulness of serial lactate monitoring in identifying patients at higher risk for surgical site complications, allowing for earlier interventions such as targeted antibiotic therapy, wound management, or additional surgical assessment.

Previous research supports this trend of rising lactate levels correlating with surgical complications. Xiaojuan et al. studied 216 gastrointestinal surgery patients, dividing them into high (Lac > 2 mmol/L) and normal lactate groups. Multiple linear regression identified surgical site and lack of intraoperative antibiotic use as risk factors for elevated lactate. Colonic surgeries had the lowest lactate levels, indicating surgical site significantly influences postoperative hyperlactatemia [8].

Following an assessment of the association between serum lactate levels and anastomotic leaks, the results showed no significant differences preoperatively (p = 0.317) or on POD 1 (p = 0.249). However, by POD 2, the p-value approached statistical significance (p = 0.064), suggesting a possible early divergence in lactate levels. By POD 3, the difference became statistically significant (p = 0.019), with the anastomotic leak group exhibiting higher lactate levels. This significance increased on POD 5 (p = 0.009) and POD 7 (p = 0.008). These findings indicate that rising serum lactate levels from POD 3 onward could serve as an early warning sign for anastomotic leaks, reinforcing the importance of close monitoring and timely intervention in high-risk patients.

These findings align with previous studies. El Rehim Hassan et al. observed in pediatric patients undergoing intestinal anastomosis that all with elevated lactate levels > 1.4 mmol/L at 12 hours developed leaks, demonstrating 100% sensitivity but only 66.7% specificity [9]. Similarly, Ip et al. studied 136 esophagectomy patients and found that elevated lactate levels on PODs 1-3 were associated with leaks. Using a Day 2 lactate cutoff of 1.7 mmol/L, they found that sensitivity and specificity were 72% and 88%, respectively. They concluded that a Day 2 serum lactate > 1.7 mmol/L should raise suspicion for a potential anastomotic leak [10].

Veličković et al. prospectively studied 195 patients undergoing major abdominal surgery, measuring lactate levels at ICU admission and at 4, 12, and 24 hours postoperatively. Among these, the 12-hour lactate level demonstrated the strongest predictive value for in-hospital mortality [11]. Similarly, Lee et al. evaluated 362 critically ill surgical patients who underwent emergency gastrointestinal surgery and found that initial hyperlactatemia (> 4 mmol/L) was significantly more common in non-survivors, with a specificity of 81.4% [12].

In line with these findings, our study also revealed a strong association between serum lactate levels and mortality. We observed statistically significant differences at all measured time points (p < 0.05). Preoperatively, patients in the mortality group had markedly higher lactate levels (5.91 mmol/L) compared to the non-mortality group (2.74 mmol/L), with a p-value < 0.001. This trend of elevated lactate persisted postoperatively, from POD 1 through POD 7. The most pronounced differences occurred on POD 5 and POD 7, with lactate levels on POD 7 reaching 4.62 mmol/L in the mortality group versus 1.27 mmol/L in survivors (p < 0.001), underscoring the prognostic value of persistent hyperlactatemia in predicting postoperative mortality.

These findings underscore the value of serial serum lactate monitoring as a reliable prognostic tool in the postoperative period. Persistently elevated lactate levels beyond POD 3 were strongly associated with SSOs, anastomotic leaks, and increased mortality, highlighting their potential as early warning markers for serious complications. Although not significantly linked to prolonged hospital stay, rising lactate levels signal the need for timely intervention, including intensified monitoring and reassessment. Incorporating routine lactate monitoring into postoperative care can aid in the early identification of high-risk patients and contribute to improved surgical outcomes.

This study had several limitations. First, we conducted this study at a tertiary healthcare facility that receives referrals, which may have introduced random errors, particularly during the sampling process. Second, the small sample size may have led to a less precise estimation of the accuracy of serial serum lactate levels. Third, being a single-center study, the results may not be generalizable to other centers specializing in surgical patient care. Finally, data collection by a single investigator may have introduced investigator bias.

Therefore, a larger multicenter study with an increased sample size and a greater number of investigators would be beneficial. 

## Conclusions

Serum lactate levels did not significantly correlate with prolonged hospital stay because there were no statistically meaningful differences between patients with and without extended hospitalization at any measured time point. However, lactate levels were significantly elevated in patients who developed SSOs and anastomotic leaks, particularly from POD 3 onward. This suggests that rising lactate levels after POD 3 could be an early warning sign for complications. Furthermore, mortality analysis revealed that persistently high lactate levels were strongly associated with an increased risk of death, with statistically significant differences observed at all measured time points. These findings highlight the potential role of serum lactate as a prognostic marker, with sustained hyperlactatemia serving as an early indicator of adverse surgical outcomes, including infection, anastomotic failure, and mortality.
